# Outcomes of pediatric patients prioritized as critical (P1/P2) by registered triage nurses from a tertiary care hospital in a low-middle-income country

**DOI:** 10.25122/jml-2024-0228

**Published:** 2025-01

**Authors:** Emad Uddin Siddiqui, Noor ul Ain, Abid Ali Jamali, Ghazala Irfan Kazi, Tooba Siddiqui

**Affiliations:** 1 Emergency Medicine Department, Aga Khan University Hospital, Karachi, Pakistan

**Keywords:** emergency department, triage category, pediatric, triage nurse

## Abstract

Triage in emergency departments (EDs) is a dynamic decision-making process to prioritize patients based on their medical care needs. The Emergency Severity Index (ESI) is a simple-to-use, five-level triage system that categorizes ED patients according to clinical urgency. The triage nurse's ability to obtain a brief history and rapidly assess clinical urgency is crucial for ensuring safe and efficient emergency care. This study aimed to assess the ability of registered triage nurses to accurately identify pediatric patients classified as Priority 1 (P1) and Priority 2 (P2) under the ESI, ensuring timely, life-saving interventions for critically ill children. A prospective chart review was conducted over six months, including all pediatric patients (aged 0–16 years) presenting to the ED and categorized as P1 or P2. Demographic data such as age, weight, gender, presenting complaints, vital signs at presentation, treatments administered in the ED, length of stay, and final disposition (admitted or discharged) were collected. Data were presented as mean ± SD or percentages with ranges. The association between the triage category and outcomes was assessed using the chi-square test. A total of 503 pediatric cases were included, with a mean age of 2.96 ± 3.79 years. The cohort comprised 311 male (62%) and 192 female patients (38%), with 250 patients (50%) under one year of age. Most patients (*n* = 308, 61%) were categorized as P2. Respiratory difficulties and fever were identified in 167 (33%) children, followed by cough/shortness of breath (SOB), fall/road traffic accident (RTA), and seizures. Among P1 cases, 11 patients (2%) required ventilatory support. A total of 384 patients (76%) were admitted to the hospital, with 297 (60%) admitted to general wards, primarily P2 cases. Significant associations were observed between triage category and outcomes, including seizures/febrile fits (P1: 20.5% vs. P2: 11.4%, *P* = 0.005) and pediatric intensive care unit (PICU) admissions (P1: 26.1% vs. P2: 5.1%, *P* < 0.001). Outcome measurements, like hospitalization, mode of admission (PICU or special care unit), mortality, length of stay in ED, cost of ED stays, and resources utilized, were all dependent on the initial triage categorization. The ESI triage system demonstrated its predictive value in determining urgency and guiding subsequent clinical decisions.

## INTRODUCTION

Emergency triage is a critical process in emergency departments (EDs) that involves prioritizing patients based on the urgency of their medical needs upon arrival. An effective triage system facilitates timely and life-saving treatment for patients with critical conditions while ensuring that all individuals requiring emergency care are assessed and categorized appropriately according to their clinical severity [1]. Many healthcare facilities implement three-level (3L) triage systems, which classify patients into three categories: emergent (Level 1), urgent (Level 2), and non-urgent (Level 3). However, studies have shown that the reliability of these systems is limited [1]. In contrast, five-level (5L) triage systems, standard practice in countries like Australia and Canada, offer greater accuracy and consistency. One of the widely recognized 5L systems is the Emergency Severity Index (ESI), developed in the United States by Wuerz and Eitel. The ESI has demonstrated superior reliability compared to 3L systems [2]. ESI is a straightforward five-level triage tool that assesses patients in the ED based on two key factors: their clinical acuity and the anticipated need for medical resources. Acuity refers to the stability of the patient’s vital signs and the potential risk to life, limb, or organ function.

The ESI is designed for use by experienced triage nurses or those who have completed dedicated training programs [3]. Accurate triage requires nurses to efficiently gather patient histories, perform focused physical assessments, and quickly determine clinical urgency. These competencies are critical for ensuring both the safety of patients and the efficiency of emergency care delivery [1].

Hospitals use triage to categorize and prioritize the patient as per their need. Institutions have different triage techniques and adopt policies per resource and patient needs. Implementing advanced systems, such as software-assisted triage, has been associated with improved outcomes [4]. The ESI has demonstrated reliability and superiority among the five-level (5L) triage systems [5]. Using vital signs as objective data in distinguishing between Priority 2 (P2) and P3 categories enhances the accuracy of ESI, making it particularly effective in resource-limited settings [6,7].

This study aimed to evaluate the efficiency of the ESI-based triage system implemented by triage nurses, ensuring that all patients requiring emergency assessment are accurately categorized according to their clinical condition. The specific objective of this study was to assess the ability of registered nurses to accurately identify P1 and P2 cases using the ESI system. This was evaluated by analyzing patient disposition from the ED to the pediatric intensive care unit (PICU), special care unit (SCU), or general wards.

### Operational definitions

Priority (P) 1: A dying patient who requires immediate lifesaving interventions.

Priority (P) 2: A patient who is in a high-risk situation or confused/lethargic/disoriented or having severe pain or distress.

## MATERIAL AND METHODS

This study was designed as a prospective chart review conducted in the emergency department of a tertiary-care hospital. Participants included all pediatric patients aged 0 months to 16 years who presented to the emergency department and were categorized as P1 or P2 during triage. The study duration was 6 months, from January 2021 to June 2021, following approval by the hospital’s Ethical Review Committee (ERC). No intervention was applied during the study.

Data collection focused on cases categorized as P1 or P2 by the triage nurses. The information recorded included demographic details (age, weight, and gender), presenting complaints, vital signs at the time of presentation, treatments received in the emergency department, length of stay in the emergency department, and the final patient disposition (admission or discharge). The level of care required (e.g., PICU or SCU) was also documented for admitted patients.

Triage was done by a senior registered nurse (RN) with at least 4 years of uninterrupted emergency room experience who completed at least a “03 Triage” course conducted biannually. Each triage team during a shift consisted of a registered nurse (RN-03), a nursing assistant (NA-01), and nursing attendants (NA-02). The team was responsible for recording vital signs, assigning triage categories, and performing re-triage as needed. They were not involved in direct patient management. To ensure accuracy and consistency, all cases categorized as P1 or P2 were reviewed and confirmed by the shift supervisor (senior staff nurse). During the six-month study period, the emergency department received 15,270 pediatric cases, of which 6.6% were either P1 or P2.

Statistical analysis was conducted using descriptive and analytical methods. Data were expressed as mean ± standard deviation (SD) or percentages with ranges. Associations between triage categories and outcomes were analyzed using the chi-square test. All statistical tests were performed as appropriate.

## RESULTS

A total of 503 pediatric participants presenting to the emergency room with either P1 or P2 were included. The mean age was 2.96 ± 3.79 years, with 311 (62%) male and 192 (38%) female patients. The majority (*n* = 250, 50%) were under one year of age, including 15% neonates (Table 1). The majority (61.2%, *n* = 308) were classified as P2 cases, with the remainder (38.8%, *n* = 195) categorized as P1.

**Table 1:** Demographic and clinical characteristics of the children

Variables	Total
*N*	503
**Age in months**	
Mean ± SD	2.96 ± 3.79
Range (Min-Max)	1 day to 16 years
**Age groups**	
Neonates	77 (15.3%)
1 to 12 months	173 (34.4%)
13 months to 2 years	55 (10.9%)
2 to 5 years	96 (19.1%)
6 to 11 years	75 (14.9%)
12 to 16 years	27 (5.4%)
**Gender**	
Male	311 (61.8%)
Female	192 (38.2%)
**Triage category**	
P1	195 (38.8%)
P2	308 (61.2%)
**Presenting complaints**	
Respiratory distress/chest congestion/difficulties in breathing (Fluctuation)	120 (23.9%)
Fever	167 (33.4%)
Cough/SOB	142 (28.2%)
Abdominal pain / Vomiting / Loose motion	75 (15%)
Seizures	64 (12.8%)
Hyperbilirubinemia / Jaundice	36 (7.2%)
Swelling and infection	18 (3.6%)
Irritability / Excessive cry	14 (2.8%)
Drowsiness	14 (2.8%)
Headache / Vertigo	13 (2.6%)
Bleeding (oral/nasal)	13 (2.6%)
Others	10 (2%)
Generalized weakness/body aches	8 (1.6%)
Rashes	2 (0.4%)
**Mechanical ventilation**	
Yes	14 (2.8%)
No	489 (97.2%)
**Stay in ED**	
< 6 hours	345 (8.6%)
> 6 hours	158 (31.4%)
**Disposition**	
Admission	384 (77.1%)
Discharge	50 (9.9%)
Left Against Medical Advice (LAMA)	61 (12.1%)
Expired in PED	3 (0.6%)
Expired in ward	5 (1%)
**Hospital stay**	***n* = 384**
PICU	48 (13.4%)
SCU	39 (10.1%)
Ward bed	297 (77.5%)

The most common presenting complaints included respiratory distress with fever (33%), followed by cough or shortness of breath (SOB), fall/road traffic accident (RTA), and seizures. Among the participants, 384 (76%) were admitted to the hospital, 297 (60%) were admitted to wards, mostly P2s, and 9.9% were discharged home. Descriptive and demographic characteristics are presented in Table 1.

Table 2 highlights the association between triage categories and patient diagnoses. Significant differences were observed between P1 and P2 cases. Seizures or febrile fits were more frequent in P1 cases (20.5% vs. 11.4%, *P* = 0.005). Respiratory distress, including pneumonia and bronchiolitis, was also more common in P1 cases (17.4% vs. 13.6%), though the association was not statistically significant. Conversely, neonatal jaundice was significantly higher in P2 cases (11.4% vs. 1%, *P* = 0.005) than acute gastroenteritis (5.5% vs. 1.5%, *P* = 0.026). Fall-related injuries and road traffic accidents (RTA) were significantly more prevalent in P1 cases compared to P2 (10.3% vs. 5.2%, *P* = 0.032), as were congenital heart defects (CHDs, 4.1% vs. 0%, *P* < 0.001). No significant differences were observed between the groups for other diagnoses.

**Table 2:** Comparison of children's diagnosis with ESI triage category (P1 & P2)

Children Diagnosis	Triage Category		*P* value
	Triage P1	Triage P2	
Total	195	308	-
Acute Appendicitis	0 [0%]	2 [0.6%]	0.260
Cardiopulmonary Arrest	1 [0.5%]	0 [0%]	0.208
Neonatal Jaundice	2 [1%]	36 [11.7%]	<0.001*
Presumed Enteric Fever / Enteric Fever	2 [1%]	8 [2.6%]	0.219
Acute Gastroenteritis	3 [1.5%]	17 [5.5%]	0.026*
Poisoning	4 [2.1%]	3 [1%]	0.315
Meningitis / Tuberculous Meningitis (TBM) / Meningoencephalitis	4 [2.1%]	8 [2.6%]	0.696
Urinary Tract Infection (UTI) / Upper Respiratory Tract Infection (URTI) / Viral Fever	7 [3.6%]	22 [7.1%]	0.096
Measles	8 [4.1%]	14 [4.5%]	0.813
Congenital Heart Disease (CHD)	8 [4.1%]	0 [0%]	<0.001*
Fall/RTA	20 [10.3%]	16 [5.2%]	0.032*
Others	20 [10.3%]	50 [16.2%]	0.059
Presumed sepsis	21 [10.8%]	19 [6.2%]	0.063
Seizures/Febrile Fits	40 [20.5%]	35 [11.4%]	0.005*
Bronchiolitis / Reactive Airway Disease / Bronchopneumonia / Pneumonia / Acute Exacerbation of Asthma / Acute Asthma / Croup / Stridor	54 [27.7%]	72 [23.4%]	0.276

Respiratory distress (*n* = 186), cough or shortness of breath (*n* = 142), and fever (*n* = 120) were the most frequent presenting complaints across both triage categories. Among the eight children who died, five presented with respiratory distress, two with cough or shortness of breath, and one with swelling or infection (Table 3).

**Table 3:** Comparison of patient dispositions based on presenting complaints

Presenting complaint	Admitted	Discharged	LAMA	Mortality
	*n* = 384	*n* = 50	*n* = 61	*n* = 8
Irritability / Excessive Cry	10 (2.6%)	1 (2%)	3 (4.9%)	0 (0%)
Fever	129 (33.9%)	16 (32%)	22 (36.1%)	0 (0%)
Respiratory Distress / Chest Congestion / Difficulties in Breathing	88 (22.9%)	10 (20%)	17 (27.9%)	5 (62.5%)
Abdominal pain / Vomiting / Loose motion	56 (14.7%)	9 (18%)	9 (14.8%)	(0%)
Hyperbilirubinemia / Jaundice	34 (8.9%)	2 (4%)	0 (0%)	0 (0%)
Generalized Weakness / Body Ache	5 (1.3%)	3 (6%)	0 (0%)	0 (0%)
Seizures	50 (13.1%)	9 (18%)	5 (8.2%)	0 (0%)
Bleeding (Oral/Nasal Bleed)	10 (2.6%)	0 (0%)	3 (4.9%)	0 (0%)
Cough/SOB	107 (27.9%)	15 (13%)	18 (29.5%)	2 (25%)
Drowsiness	11 (2.9%)	2 (4%)	0 (0%)	(0%)
Swelling and infection	13 (3.4%)	1 (2%)	3 (4.9%)	1 (12.5%)
Headache / Vertigo	10 (2.6%)	1 (2%)	2 (3.3%)	0 (0%)
Rashes	2 (0.5%)	0 (0%)	0 (0%)	0 (0%)
Others	6 (1.6%)	2 (4%)	2 (3.3%)	0 (0%)

Significant differences were observed in mechanical ventilation requirements between triage classifications when categorizing patients using ESI. A total of 12 patients (6.2%) in the P1 category required mechanical ventilation compared to only two patients (0.6%) in the P2 category (*P* < 0.001). Differences in patient disposition were also significant (*P* = 0.005). Mortality was higher in the P1 group (4.1%) compared to no deaths in the P2 group. Conversely, the likelihood of discharge was higher in P2 cases (11.7%) compared to P1 cases (7.1%). However, there were no significant differences between the groups regarding hospital admission rates. Additionally, no differences were observed between P1 and P2 groups regarding age, gender, or length of stay in the pediatric emergency department (PED) (Table 4).

**Table 4:** Baseline and clinical characteristics of pediatric patients compared by triage

Baseline and clinical characteristics	Triage category		*P* value
	Triage P1	Triage P2	
Total	195	308	-
**Age groups**			
Neonates	23 [11.8%]	54 [17.5%]	0.192
1 to 12 months	72 [36.9%]	101 [32.8%]	
13 months to 2 years	26 [13.3%]	29 [9.4%]	
2 to 5 years	39 [20%]	57 [18.5%]	
6 to 11 years	23 [11.8%]	52 [16.9%]	
12 to 16 years	12 [6.2%]	15 [4.9%]	
**Gender**			
Male	123 [63.1%]	188 [61%]	0.647
Female	72 [36.9%]	120 [39%]	
**Mechanical ventilation**			
Yes	12 [6.2%]	2 [0.6%]	<0.001*
No	183 [93.8%]	306 [99.4%]	
**Stay in PED**			
<6 hours	132 [67.7%]	213 [69.2%]	0.730
>6 hours	63 [32.3%]	95 [30.8%]	
**Disposition**			
Admit	149 [76.4%]	235 [76.3%]	0.005*
Discharge	14 [7.2%]	36 [11.7%]	
Lama	24 [12.3%]	37 [12%]	
Expired in PED	3 [1.5%]	0 [0%]	
Expired in ward	5 [2.6%]	0 [0%]	
**Stay in hospital**			
PICU	40 [26.1%]	12 [5.1%]	<0.001*
SCU	16 [10.5%]	23 [9.8%]	
Ward bed	97 [63.4%]	200 [85.1%]	

## DISCUSSION

The increasing influx of patients in emergency rooms significantly disrupts the normal functioning of healthcare systems worldwide [8]. Triaging serves as a critical initial assessment process at the entrance of an emergency department, enabling the rapid and safe identification of the sickest patients for prioritized care. Our findings revealed several key observations. Approximately one-third of patients experienced delayed dispositions of more than 6 hours. Notably, 7% of P1 cases were discharged directly from the ED, highlighting potential ambiguities or technical errors in their initial categorization. Additionally, around 3% of patients were inappropriately transferred to non-monitored ward beds, raising concerns about disposition accuracy. Among patients categorized as critical, the majority (30%) presented conditions such as sepsis, seizures, or febrile illness.

Different institutions have different triage techniques and adopt policies per their needs; however, they prioritize clinical urgency based on the patient’s need. This study aimed to evaluate the accuracy of registered nurses in identifying P1 and P2 cases using the ESI system and ensuring the appropriate disposition of patients from the emergency department to hospital areas such as the PICU, SCU, or general ward. Our findings revealed that most patients were under 12 months of age and were predominantly categorized as P2 based on the ESI severity index. In terms of clinical presentation, most children presented with fever, cough, and respiratory distress, and nearly all required hospital admission.

ESI appears to be the most reliable among all five-level triage systems, demonstrating excellent inter-rater agreement (kappa 0.84–1) [5]. However, ESI has also exhibited the highest percentage of under-triage [6]. Objective data, such as vital signs—especially in the P2 and P3 categories—makes the system more sophisticated and reliable in identifying critical patients early. However, performing triage and assessing children in emergencies is always more challenging than for adults. Studies indicate that children require special considerations during the triage assessment process due to their developmental, epidemiological, and physiological variations across different age groups [9-12].

Timely decision-making in critical cases during busy emergency department hours is crucial for reducing morbidity, mortality, and ED length of stay, and ensuring proper patient disposition. Errors in triage can have significant consequences: under-triage may delay time-critical interventions and decisions, while over-triage can overwhelm ED resources, disrupt patient flow, and prolong the triage process, potentially leading to adverse patient outcomes [9]. Achieving accuracy in triage requires both learning and experience, particularly in distinguishing between P2 and P3 categories. This distinction often relies on objective data, such as the documentation of vital signs. Furthermore, the accuracy of triage decisions is also contingent upon the nature and quality of the necessary data obtained by the nurse to decide acuity [13]. Trauma care is highly time-sensitive, and the early identification of potentially fatal injuries and conditions is crucial for survival [9]. Despite this critical need, most of the existing literature on emergency triage focuses on non-trauma cases in hospital settings. Studies specifically addressing emergency triage among non-trauma pediatric patients or pre-hospital settings are relatively few [14,15].

The data from our study center indicates a notably high proportion of P1 and P2 cases, accounting for approximately 10% of the total cases (based on nursing data from the study center). This elevated percentage may be attributed to several factors, including limited availability of primary healthcare services, delays in accessing tertiary care facilities, and socioeconomic constraints.

One limitation of our study is the exclusion of P3 cases. This creates a potential gap, as patients initially categorized as P3 may later require upgrading to P2 based on their evolving clinical status. These cases were not captured, which may have impacted the comprehensiveness of our findings. We recommend that future studies include P1 to P3 cases using the ESI triage tool. Additionally, comparative research evaluating the efficacy of ESI and other triage tools or scoring systems is essential to enhance triage accuracy and optimize outcomes.

## CONCLUSION

Outcome measurements such as hospitalization rates, mode of admission (PICU/SCU), mortality, length of stay in the ED, cost of ED stays, and resource utilization heavily depend on initial triage categorization. As expected, the findings demonstrate that P1 patients are more likely to require advanced interventions such as mechanical ventilation and intensive care (PICU). In contrast, P2 patients, although less critical, still demand significant resources. Previous studies highlight the importance of redesigning and modifying triage systems for optimal categorization and appropriate patient disposition. Using outcome measurements, such as admission rates via the ESI triage system, may help predict the mode of admission and demonstrate a strong correlation between clinical urgency and other variables.
